# Stochasticity and Spatial Interaction Govern Stem Cell Differentiation Dynamics

**DOI:** 10.1038/srep12617

**Published:** 2015-07-31

**Authors:** Quinton Smith, Evgeny Stukalin, Sravanti Kusuma, Sharon Gerecht, Sean X. Sun

**Affiliations:** 1 Department of Chemical and Biomolecular Engineering, Johns Hopkins University, Baltimore, MD 21218.; 2 Department of Mechanical Engineering, Johns Hopkins University, Baltimore, MD 21218.; 3 Johns Hopkins Physical Sciences-Oncology Center, Johns Hopkins University, Baltimore, MD 21218.; 4 Department of Biomedical Engineering, Johns Hopkins University, Baltimore, MD 21218.; 5 Department of Material Science and Engineering, Johns Hopkins University, Baltimore, MD 21218.

## Abstract

Stem cell differentiation underlies many fundamental processes such as development, tissue growth and regeneration, as well as disease progression. Understanding how stem cell differentiation is controlled in mixed cell populations is an important step in developing quantitative models of cell population dynamics. Here we focus on quantifying the role of cell-cell interactions in determining stem cell fate. Toward this, we monitor stem cell differentiation in adherent cultures on micropatterns and collect statistical cell fate data. Results show high cell fate variability and a bimodal probability distribution of stem cell fraction on small (80–140 μm diameter) micropatterns. On larger (225–500 μm diameter) micropatterns, the variability is also high but the distribution of the stem cell fraction becomes unimodal. Using a stochastic model, we analyze the differentiation dynamics and quantitatively determine the differentiation probability as a function of stem cell fraction. Results indicate that stem cells can interact and sense cellular composition in their immediate neighborhood and adjust their differentiation probability accordingly. Blocking epithelial cadherin (E-cadherin) can diminish this cell-cell contact mediated sensing. For larger micropatterns, cell motility adds a spatial dimension to the picture. Taken together, we find stochasticity and cell-cell interactions are important factors in determining cell fate in mixed cell populations.

Cell phenotypic dynamics govern a variety of critical physiological processes ranging from organismal development, to cancer/disease biology, and tissue regeneration. Starting from undifferentiated pluripotent stem cells (PSCs), subsequent differentiation and developmental processes have been explored in many settings, dating back to the Waddington Landscape^1^. Recently, multiple experiments have shown that differentiated cells can return to the pluripotent state and interconvert to other types of differentiated cells^2^,^3^,^4^. However, quantitative understanding of factors influencing differentiation decisions is still lacking. Developing mathematical models would allow us to quantitatively predict cellular compositions over time in different types of environments. In the present paper, we focus on understanding and quantifying the role of cell-cell interactions in stem cell fate determination. We examine differentiation dynamics of human induced PSC (hiPSCs) in confined adherent cultures on micropatterns of varying sizes (80–500 μm, Fig. 1). Many replications of cell cultures in identical conditions are analyzed to obtain statistical information. We find that mesoderm stem cell differentiation is highly stochastic, and quantitatively described by a probabilistic model. From the data, we are able to discern the differentiation probability as a function of the local stem cell fraction and microenvironment. Results show that stem cells surrounded by differentiated cells will differentiate faster; undifferentiated status is more likely maintained when stem cells only interact with other stem cells. This cell-cell interaction governing differentiation can be partially blocked by interfering with E-cadherin. We show that this cell-cell interaction, coupled with cell motility, can generate dynamic spatial patterns of stem and differentiated cells on larger micropatterns.

To examine mesoderm differentiation dynamics, we utilized a previously established adherent culture differentiation scheme, which directs hiPSCs towards vascular lineages^5^,^6^ and followed the expression of a pluripotency marker after 1, 2 and 5 days in culture. By systematically changing the cell substrate size and exchanging differentiation media daily, we can control the spatial extent of cell-cell interactions while limiting cytokine-mediated responses. For example, on small 80 μm micropatterns, there are at maximum, 3–5 cells. Since cells can move freely within the pattern any individual cell is in contact with all other cells. In contrast, on large 500 μm micropatterns, cells can only explore their immediate neighborhood within the first day of differentiation. While previous studies have examined the effects of micropattern size on stem cell differentiation^7^,^8^,^9^, they have been limited in exploring osteo/adipogenic potential in heterogeneous mesenchymal stem cell populations. Using a similar micropatterned array we have recently shown the role of confinement in lineage specification of differentiating vascular cells^10^, however incorporating an additional layer of computational based predictive models will lead to higher differentiation efficiency and understanding of fate decision. Although modeling stem cell population dynamics^11^,^12^,^13^,^14^,^15^ including feedback and feed-forward mechanisms in tissues based on mean-population models have been explored^16^,^17^, stochastic population models based on constant differentiation probability have only been applied in epidermal stem cell differentiation^18^,^19^. Here we focus on exploring stochastic behavior in homogeneous stem cell populations during the loss of pluripotency, where large variations in population composition are observed. Since our differentiation conditions consistently induce vascular lineages, the stochasticity is not about variations in the final cell type. Rather, the stochasticity is inherent in the population dynamics during the mesoderm differentiation process. Experimentally obtained statistical distributions of pluripotent and differentiated cell types allowed us to develop a quantitative probabilistic model to understand stem cell differentiation kinetics. We demonstrate that the stochastic differentiation model gives qualitatively different results than a mean population model. The model is able to explain the bimodal probability distribution of stem cell fractions seen on small micropatterns, and can also predict average cell populations in the micropatterns over time as well as statistical fluctuations of the population. The simple generalization of the model that introduces spatial compartments can also explain results on larger micropatterns. Cell-cell interaction together with cell motility can develop spatial cell patterns on larger substrates, which may explain patterns seen in other developmental systems.

## Results

### Stem cell differentiation dynamics on spatially confined culture substrates

**Figure 1:**
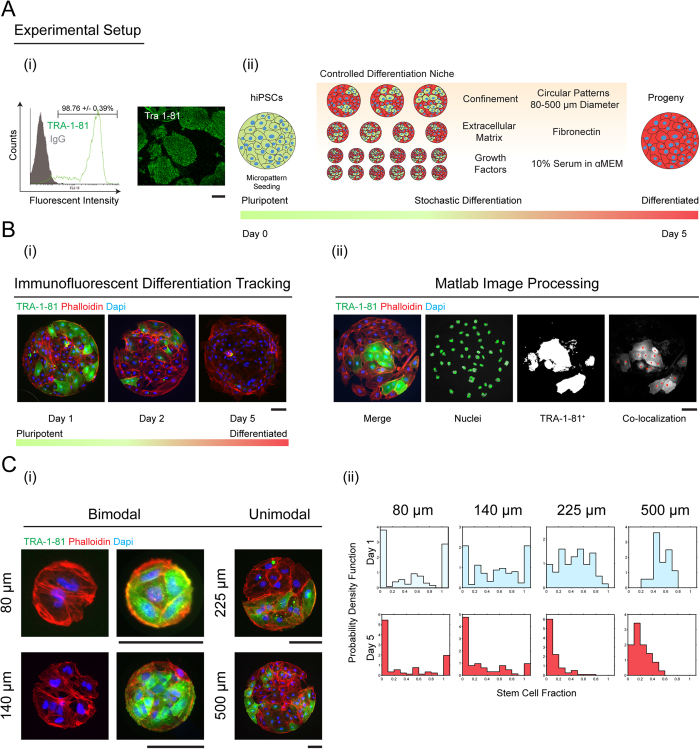
Homogenous hiPSC population matured on circular micropatterns show non-homogeneous differentiation dynamics depending on the size of confinement. (**A**) (i) hiPSCs are plated on fibronectin coated circular substrates ranging from 80–500 μm in diameter. Cell seeding density is 100,000 per coverslip. The initial cell hiPSC population is 98.67 +/− 0.39% pluripotent as demonstrated by TRA-1-81 flow cytometry and staining data. (ii) Hundreds of identical micropatterns are replicated in the same culture. Cells grow and differentiate for 5 days. (**B**) (i) Differentiation demonstrated by loss of green intensity (ii) The cell culture is fixed and stained at regular intervals and images are processed and quantified for each micropattern. The number of stem and differentiated cells are recorded to obtain population distributions. The image analysis algorithm is discussed in the SM. (**C**) (i) Representative images of stem cell populations grown for 1 day on circular micropatterns. (ii) Probability density functions of stem cell fractions quantified from (i) showing bimodal probability distributions of stem cells on smaller (80 and 140 μm) and unimodal distributions on larger (225 and 500 μm) diameter micropatterns. The 80 and 140 μm micropatterns show that it is very probable to observe a micropattern with 100% stem cells or 100% differentiated cells. For the larger 225 and 500 μm micropatterns, the opposite is true (TRA-1-81 in green; phalloidin in red; nuclei in blue; scale bars are 100 μm).

In our established step-wise differentiation strategy we observe an increase in mesodermal gene expression, stagnant neuronal expression, and loss of pluripotency six days post differentiation. To elucidate the kinetics of mesoderm induction, we differentiated hiPSCs using our established protocol^6^,^20^ on identical 2D fibronectin circular micropatterned surfaces (Fig. 1A and SM) ranging from 80–500 μm in diameter, and tracked the loss of pluripotency. In comparison to mesencyhmal stem cells, hiPSCs are advantageous in studying differentiation dynamics in that there is very little heterogeneity in the starting population (SM Fig. S2). With this system, we are able to quantify the differentiated state on tens to hundreds of identical culture conditions, differing only in pattern size (Materials and Methods). Undifferentiated stem cells were identified by immunostaining for pluripotent marker, tumor rejection antigen 1-81 (TRA-1-81), and the total cell number was enumerated by staining the cell nuclei (Fig. 1Bi). For each circular micropattern, we obtained the number of undifferentiated stem cells and differentiated cells after 1, 2 and 5 days of culture using a custom MATLAB image processing program (Fig. 1Bii).

Results from these quantitative experiments show a striking feature. Under mesoderm inducing differentiation media, for small 80 and 140 μm micropatterns, it is highly likely to observe either 100% stem cells or 100% differentiated cells (Fig. 1Ci). Here, the stem cell fraction for each pattern is defined as *χ* = *n*_*s*_/(*n*_*s*_ + *n*_*D*_), where *n*_*s*_ and *n*_*D*_ are the number of stem cells and differentiated cells on each pattern, respectively. Experimentally, the fraction of stem cells is also the percentage of cells expressing pluripotent marker TRA-1-81 in each of the analyzed micropatterns. The total population for each pattern is counted from DAPI nuclear staining and the percentage or fraction of TRA-1-81 positive cells is recorded as *χ*. This observed distribution of stem cells can be characterized as bimodal, i.e., it is strongly peaked at *χ* = 0 and *χ* = 1 (Fig. 1Cii). The probability of observing some mixture of stem and differentiated cells is smaller. In contrast, for larger 225 and 500 μm substrates, the probability distribution shows a qualitatively different behavior: it is highly likely to observe micropatterns with a mixture of undifferentiated and differentiated cells (Fig. 1C). Consistent with unconfined differentiation, the probability of observing differentiated cells increases over time, and by day five, a large fraction of micropatterns have differentiated completely (Fig. 1Cii, bottom), although a large variability from pattern to pattern still remains. Although the influence of microenvironment size on stem cell differentiation has been noted before^17^, statistical information such as presented in Fig. 1, has not been reported in a controlled adherent differentiation scheme. While each culture micropattern of a particular size is identical, except for some variations in the initial number of seeded cells, these results show that the kinetics of loss of pluripotency during differentiation is highly stochastic. Therefore, stem cells do not execute deterministic responses to microenvironmental signals. Within this system, stem cell differentiation decisions are probabilistic and the population behaves stochastically, as noted in other contexts such as differentiation in the intestinal crypt^21^,^22^.

### Model for understanding stem cell differentiation dynamics

**Figure 2:**
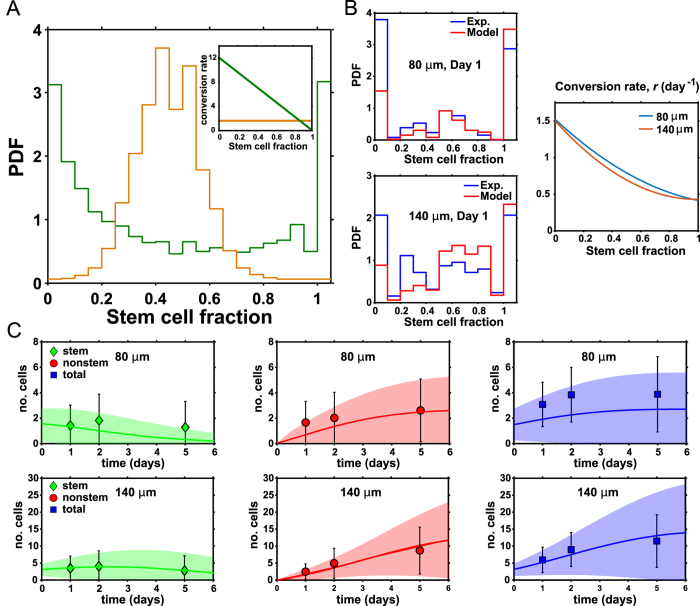
Model and experimental comparison. (**A**) Quantitative modeling shows that the form of the differentiation transition probability, Eq. (2), affect the stem cell fraction distribution. From Eq. (1), if the differentiation probability *r*, is a constant (brown line), then the stem cell fraction distribution only shows a single peak around 50% (brown histogram). In contrast, if *r* is a declining function of local stem cell fraction in the micropattern, i.e., differentiation is more likely when pluripotent cells are surrounded by differentiated cells (green line), then the stem cell population shows bimodal behavior (green histogram). (**B**) Comparisons of experimental probability density function of stem cell fraction (blue) with mathematical model results (red). The form of the stem cell differentiation probability, *r* in Eq. (2), that best explain the experiment is also shown. This function is relatively independent of the micropattern size, which is consistent with modeling assumptions. (**C**) The model also explains the average populations of stem and differentiated cells, as well as population fluctuations. The shaded region represents the range of population fluctuation, defined by the computed standard deviation. The computed average population is the solid line and the data are symbols with measured standard deviation.

In order to reconcile the disparate stem cell fraction distribution observed for different micropattern sizes, we consider a stochastic population model where stem cell growth and differentiation events are governed by transition probabilities (Fig. 2). There is also some probability of cell loss from detaching from the substrate. The central quantity in this minimal model is the probability of observing a particular number of stem and differentiated cells at time *t* on each micropattern: *P*(*n*_*s*_, *n*_*D*_, *t*), where *n*_*s*_ and *n*_*D*_ are the numbers of stem and differentiated cells on each micropattern, respectively. This probability density evolves in time according to a master equation (or fundamental conservation of probability)^23^,^24^:


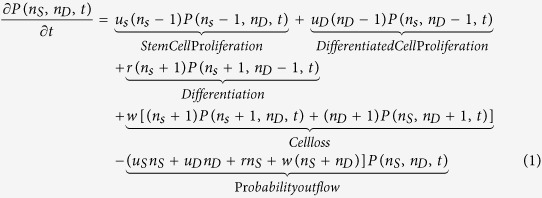


where *u*_*S,D*_ are the symmetric cell division rates for stem cells and differentiated cells, ***r*** is the differentiation conversion rate from stem to differentiated cells, and *w* is the rate of cell loss. The quantity we will try to clarify in this paper is that the differentiation rate, ***r***, is not a constant but depends on the character and composition of the local cell culture environment, i.e., *r* ∫ *r*(*n*_*S*_, *n*_*D*_). The cell division rates, *u*_*S,D*_, are also not constants and depend on the cell population size. This is because there is cell-cell competition and each micropattern can support a finite number of cells (see SM text). For smaller 80 and 140 μm micropatterns, cells are able to move sufficiently to make contact with other cells in the same culture micropattern. Therefore, dynamics governed by Eq. (1), which describes a well-mixed population of stem and differentiated cells, is reasonable. From these cell-cell interactions, the signaling pathway governing stem cell differentiation obtains information about the composition of the local cell neighborhood and makes decisions accordingly.

### Stem cell-mediated inhibition of differentiation

Starting with Eq. (1), for given functional forms of ***r***, *u*_*S,D*_ and *w*, and an initial cell number distribution *P*_0_(*n*_*S*_, *n*_*D*_), we can compute the theoretical number of stem and differentiated cells (see SM). The initial cell number distribution is Poissonian, and is given by the seeding density (100,000 per slide, see SM). We also do not assume confluent cell number in each patch. The computational analysis shows that the functional form of ***r*** has a strong influence on the distribution of stem cell fractions (Fig. 2). For instance, in a well-mixed system, the differentiation rate could be a function of the fraction of stem cells: *χ* = *n*_*S*_/(*n*_*S*_ + *n*_*D*_).

From our experimental data, we reason that ***r*** must be a declining function of the stem cell fraction (Fig. 2): if there are large fractions of stem cells, then differentiation is less likely. If there are large fractions of differentiated cells, then the remaining stem cells will differentiate faster. This type of differentiation probability is sufficient to generate the bimodal probability distribution seen in 80 and 140 μm micropatterns (Fig. 1). Computational analysis confirms that in order to generate the bimodal probability distributions of stem cell fractions seen in Fig. 1, *r* must be a declining function of χ. If *r* is independent of χ, then the stem cell fraction distribution is unimodal (Fig. 2A and SM). Additionally, quantitative fitting to the data suggest that the differentiation probability is a slight nonlinear function of *χ*, i.e.,


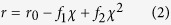


where *r*_0_ is the differentiation rate at zero stem cell fraction and (*f*_1_, *f*_2_) are constants. This result is not surprising since signaling pathways governing differentiation likely contain cooperative behavior, probably in the form of Hill functions that generates switch-like behavior. The molecular details of the signaling pathway are still unclear and require further investigation.

### Model predictions of long-term growth dynamics on small micropatterns

As time progresses, experiments show that the proportion of stem cells and differentiated cells change. The total cell population also generally increases (Fig. 2C), features captured by our model (Fig. 2C). The total population change depends on cell division, cell loss, and differentiation rates. Since cells are grown on finite size micropatterns, the total cell population must be limited. Cells must compete for space, and therefore *u*_*S*_ and *u*_*D*_ also depend on the current population size. We use a logistic growth type of expression to model this, i.e., *u*_*S*_ = *v*_*S*_ − *γ*_*S*_(*n*_*S*_ + *n*_*D*_ − 1) and *u*_*D*_ = *v*_*D*_ − *γ*_*D*_(*n*_*S*_ + *n*_*D*_ − 1) where *v*_*S,D*_ are growth rates in absence of competition (total cell number is zero) and *γ*_*S,D*_ are competition or crowding parameters. For cell loss, no visible cell death occurred during our experiment. However, some cells do detach from the substrate. Therefore we consider a constant cell loss rate, *w*.

We considered three different modes of stem cell division in our model to capture their unique properties of self-renewal and differentiation. Discussions on possibilities of symmetric and asymmetric cell division, or direct conversion without division in stem cells have been extensively reviewed^25^. The notion of asymmetric stem cell division via cell polarity has been extensively examined, with highly conserved mechanisms between vertebrates and invertebrates. The primary evidence of asymmetric stem cell differentiation relies on the well-studied Drosophila germ stem cell niche. However adult stem cells have been shown to exhibit this ability as well^26^,^27^.

Within Eq. (1), the first term in the equation describes symmetric cell division where *n*_*S*_ − 1 stem cells is replaced by *n*_*S*_ stems cells and *n*_*D*_ remains unchanged. The third term describes direct conversion where *n*_*S*_ + 1 stem cells and *n*_*D*_ − 1 differentiated cells is replaced by *n*_*S*_ stem cells plus *n*_*D*_ differentiated cells. The relative probability of symmetric division versus direct conversion depends on the ratio between *u*_*S*_ and ***r***. We can also consider asymmetric cell division, i.e., a stem cell divides to form a stem cell and a differentiated cell. In this case, the third term would be replaced by *rn*_*S*_*P*(*n*_*S*_, *n*_*D*_ − 1, *t*) and other terms remain the same. Notice that with asymmetric cell division, the number of stem cells generally does not decrease. Under our controlled differentiation scheme we expect a complete loss of stem cells, evidenced by our experimental data. As a result we considered direct stem cell conversion in our system. Stem cells could also divide to form two differentiated cells. In this case, the third term would modify slightly to *r*(*n*_*S*_ + 1)*P*(*n*_*S*_ + 1, *n*_*D*_ − 2, *t*). We find that the solution of the equation is essentially the same for this case. It is also possible that asymmetric cell division and direct conversion are occurring simultaneously. Our results cannot distinguish this possibility, but adding this complication does not change the qualitative picture, especially the obtained form of the differentiation probability, ***r*** in Eq. (2).

### Average cell population and fluctuations in cell composition

Eq. (1) is able to predict not only the average population in the micropatterns, but also the variation (fluctuation) in populations of different types of cells. The average populations are computed as:


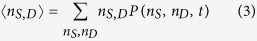


The population fluctuations are given by 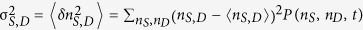. The comparisons between model predictions and experimental data are shown in Fig. 2C. We see that the expected population fluctuations are generally well captured by the stochastic model. In the SM, we also discuss a mean population model, which can compute the average cell populations. However, the mean population model is quantitatively different from the stochastic model, and cannot compute population fluctuations or distributions of stem cell fractions.

### Cell motility plus differentiation forms spatial patterns

**Figure 3:**
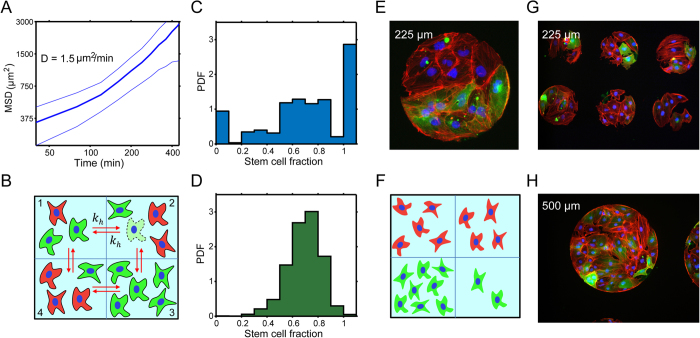
Cell motility and cell-cell interaction can explain spatial patterns seen on larger micropatterns. (**A**) Measured means squared displacement versus time for stem and differentiated cells, giving a diffusion constant of 1.5 μm^2^/min. (**B**) A compartment model for large micropatterns. A 225 μm pattern can be viewed as four, 80 μm patterns connected together. Cells within each compartment are well mixed and interact with each other. Cells can also migrate between adjacent compartments, modeled by stochastic hopping rates *k*_*h*_. (**C**) Computed stem fraction probability distribution for a single compartment within the large micropattern. The compartment shows the same identical bimodal behavior as the smaller micropatterns. (**D**) Computed stem cell fraction distribution for the large micropattern when four compartments are summed. This distribution is unimodal, in accord with observations in Fig. 1C. (E) An example immunofluorescence image showing spatial domains within the 225 μm micropattern. Lower half are dominated by stem cells. (**F**) A sample simulated 225 μm micropattern, showing similar micro domains dominate by stem cells. The simulations are performed using a Gillespie algorithm described in the SM. Additional examples of immunofluorescence images of spatial patterns seen (**G**) on 225 μm and (**H**) 500 μm micropatterns after one day of differentiation. (TRA-1-81 in green; phalloidin in red; nuclei in blue; scale bars are 100μm).

For the larger 225 and 500 μm culture micropatterns, global stem cell fractions collected over the whole micropattern no longer exhibit bimodal behavior (from Fig. 1). One possibility is that the well-mixed assumption in Eq. (1) is no longer valid and cells do not explore the complete micropattern. We hypothesize that differentiation is governed by local cell composition, and on larger micropatterns cells are unable to move sufficiently to sample the whole space. To test this, we recorded time-lapse movies of differentiating stem cells on these micropatterns and collected mean squared displacement data (Fig. 3A). We tracked single cells over 24 hrs and measured their averaged mean squared displacement. We find that cells generally move randomly and travel about 100 μm over 24 hrs. This translates to a diffusion coefficient of ∼1.5 μm^2^/min. The time scale of diffusion over 100 μm is comparable to the time scale of cell division and differentiation. We incorporate cell spatial diffusion using a simple extension of our model in Eq. (1). For the 225 μm micropattern, we model it as four connected compartments, each about 100 μm in size (Fig. 3B). Within each compartment, cells divide and differentiate according to the local composition of the compartment. In addition, cells can move from compartment to compartment according to stochastic transitions from random diffusion. This model can completely explain the observed histograms for 225 and 500 μm micropatterns (Fig. 3). Within each compartment, the stem cell composition still follows the bimodal distributions seen in smaller micropatterns; but when the compartments are summed to generate the total histogram for the larger micropattern, the total histogram reflects an average of the compartment histograms. From simulations, we see that individual compartments can still be dominated by either stem cells or differentiated cells. This is also seen in experimental images (Fig. 3), where we can observe spatial domains of differentiated and pluripotent cells.

From results of the smaller micropatterns, the form of the differentiation probability, ***r***, suggests that cell-cell spatial interaction is important. Therefore, together with cell diffusion, spatial patterns can form in differentiating populations of stem cells. If stem cells are surrounded by other stem cells, the probability of differentiation is low. If differentiated cells surround a stem cell, then it is more likely for stem cells to differentiate. From our experiments, it is possible to see these spatial domains on larger micropatterns (Fig. 3). These micropatterns often show a localized region where stem cells are clustered, approximately 100 μm in size. The spatial patterns observed in our experiment are not Turing-like patterns, which typically arise from substantially different diffusion constants of different species. Here, stem and differentiated cells have a similar diffusion constant. Rather, these patterns arise from cell-cell spatial coupling and the nonlinear nature of the differentiation probability. These spatial patterns are also reminiscent of patterns seen in differentiation on similar micro domains in the presence of BMP4^26^. Mathematical modeling using a mean population approximation of the stochastic master equation in the spatial regime also cannot capture these patterns (see SM). These patterns appear naturally from fluctuations and stabilization of fluctuations due to cell-cell interaction.

### The role of E-cadherin in stem cell differentiation

**Figure 4:**
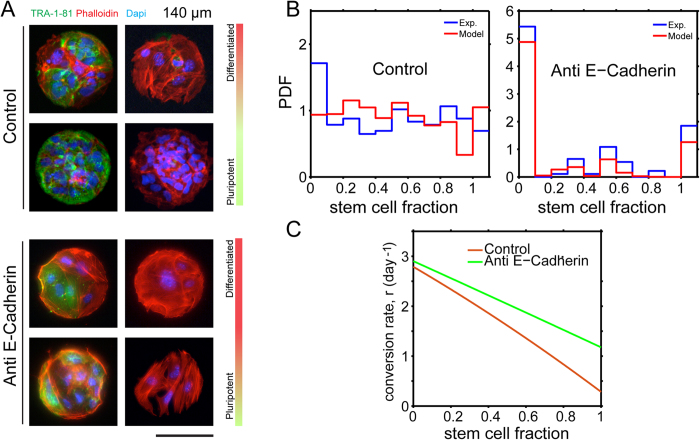
Uncoupling the role of cell-cell contact and differentiation in micropatterns with E-cadherin antibody treatment. Cell seeding density is 500,000 per cover slip. (**A**) In the presence of E-cadherin antibody, the fraction of differentiated cells increases after 24 hrs in culture on 140 μm diameter patterns. (**B**) Quantitative analysis shows that E-cadherin changes differentiation kinetics so that a larger percentage of cells are differentiating when compared to the control. (**C**) Modeling results show that the differentiation probability, *r* in Eq. (2), has changed significantly. In the control experiment, the differentiation probability at 100% stem cell fraction is less than 3 times the probability at 0% stem cell fraction. With the addition of an E-cadherin antibody, the differentiation probability at 100% stem cells is substantially higher.

E-cadherin is essential in not only maintaining cell-cell interactions, but also pluripotency in stem cells. To elucidate this role of cell-cell interactions in stem cell differentiation kinetics within confined geometric domains, 50 μg/mL of anti-E-cadherin antibody (clone 67A4; Millipore) was incubated with freshly dissociated hiPSCs for 2 hrs^28^. As evidence suggests disruption of E-cadherin signaling leads to increased stem cell death^29^, 500,000 cells were subsequently seeded onto the micropatterns, cultured for an additional 24 hrs, prepped for immunofluorescence, and subsequently analyzed for differentiation kinetics. We see that treated cells on 140 μm domains after one day exhibited qualitatively different behavior than the control experiment. There is a significantly larger portion of differentiated cells when E-cadherin is inhibited (Fig. 4A). Quantitative analysis of the data shows that the differentiation probability, *r*, is a flatter function of stem cell fraction when E-cadherin is blocked (Fig. 4B). At χ = 1, for example, the differentiation probability in the presence of anti E-cadherin antibody is more than two times larger than the control. At χ = 0, the differentiation probabilities are nearly identical. Similar results are also observed for 80 μm domains. For larger domains, the spatial patterns are also significantly different in the presence of anti E-cadherin when compared with the control. These results suggest that when E-cadherin is blocked, a stem cell surrounded by other stem cells no longer recognizes the local environment effectively, and differentiates with a similar probability as when it is surrounded by differentiated cells (χ = 0). Note that even with anti E-cadherin, *r* as a function of χ is not completely flat, suggesting that stem cells are still recognizing their neighbors to some extent. This could be due to inefficient E-cadherin blocking through antibody incubation or that other independent signaling mechanisms allow for cell-cell interactions. Taken together, we conclude that cell-cell interaction, partially mediated by E-cadherin related cell signaling, is critical in regulating the stem cell early differentiation events. Note that the inhibition of E-cadherin also influences cell proliferation parameters. The actual observed population distributions result from a combination of these parameters.

## Discussion and Conclusions

By analyzing stem cell differentiation dynamics in many spatially defined microenvironments, we found strong stochastic behavior during the differentiation process. The composition of individual micropatterns varied dramatically over the time course of the differentiation. On smaller micropatterns, we observe that the most probable composition is either 100% stem cells or 100% differentiated cells. Moreover, the physical dimensions of the microenvironment can influence stem cell differentiation in significant ways. We propose a stochastic differentiation model frame-work, and showed that stem cell differentiation probability is a strong function of local stem cell fraction within the immediate cell vicinity. When stem cells are surrounded by other stem cells, the differentiation decision is slow; whereas, when differentiated cells surround stem cells, then the differentiation rate is faster by nearly three fold. This result is consistent with the previous proposal that there are feedback signals between differentiated cells and stem cells^16^. The proposed stochastic modeling framework should be applicable in other settings for understanding differentiation dynamics.

We also found that the cell-cell interaction during differentiation is partially mediated by an E-cadherin governed signaling mechanism. Although, cell-cell interaction is not completely inhibited in our experimental conditions, we are able to manipulate, observe, and quantify variances in differentiation kinetics when the roles of cell contact in spatially confined domains are altered. It is possible that E-cadherin affects multiple sensing mechanisms in stem cells and there are redundant mechanisms that reinforce cell-cell interaction in stem cell niches.

For larger micropatterns, we see that because differentiation decisions are mediated by cell-cell interaction and cell motility is on a similar time scale as cell differentiation, this leads to coarsening and spatial pattern formation where domains of stem and differentiated cells appear. Similar observations of these stem cell patterns have appeared recently for *in vitro* systems^30^. Mathematically, the domain size is governed by cell motility and the dependence of differentiation rates on local cell composition. We believe that the type of spatial patterns is driven by stochastic fluctuations, and cannot be captured by mean population of models. The patterns are also not consistent with the Turing-type, where differential diffusion constants of multiple species play a major role in establishing pattern length scales^31^. Rather, the patterns are driven by nonlinearities in the kinetic rate parameters.

These results suggest that stem cell differentiation is reminiscent of dynamics seen during phase transitions in mixed systems. Here, stem cell differentiation is partially nucleated by existing differentiated cells. Therefore, domains of differentiated cells will likely grow. However, cell motility and diffusion is relatively slow, therefore domains do not grow from coalescence of smaller domains. For systems with complex geometries, and/or changing microenvironments, more complicated spatial patterns may appear, and can even exist as steady state configurations. Therefore, cell-cell interaction may explain spatial patterns of differentiation and growth seen during developmental processes. Further quantitative studies may allow us to manipulate patterns by modifying these interactions.

## Additional Information

**How to cite this article**: Smith, Q. *et al.* Stochasticity and Spatial Interaction Govern Stem Cell Differentiation Dynamics. *Sci. Rep.* **5**, 12617; doi: 10.1038/srep12617 (2015).

## Supplementary Material

Supplementary Information
