# Supplementary material for: Stochasticity and Spatial Interaction Govern Stem Cell Differentiation Dynamics
Source: Sci Rep. 2015 Jul 31;5:12617. doi: 10.1038/srep12617 (PMC4521170; doi:10.1038/srep12617)
Supplement: Supplementary Information [file srep12617-s1.pdf]

Stochasticity and Spatial Interaction Govern Stem Cell Differentiation Dynamics: Supplemental Material

Quinton Smith[1], Evgeny Stukalin[2], Sravanti Kusuma[1], Sharon Gerecht[1,4] and Sean X. Sun[2,3,4,1]

[1]*Department of Chemical and Biomolecular Engineering,* [2]*Mechanical Engineering,* [3]*Biomedical Engineering and* [4]*Johns Hopkins Physical Sciences-Oncology Center, Johns Hopkins University, Baltimore, MD 21218, USA*

I. MATERIALS AND METHODS

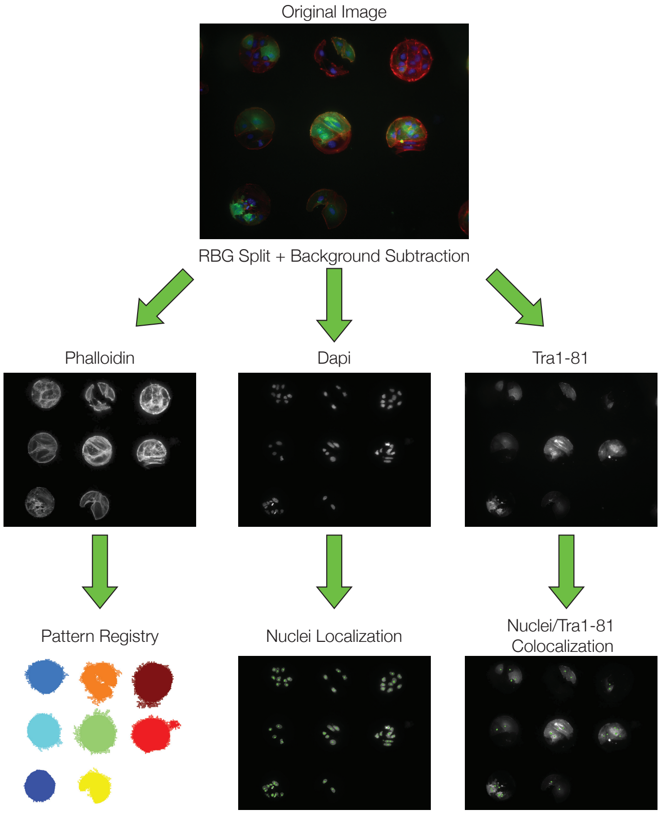

FIG. S1. Graphical representation of computational image analysis algorithm. From an image of stained micro patterns where Phalloidin is in red, Dapi is in blue and Tra1-81 is in green, individual channels are separated and processed using a Matlab image processing algorithm described below.

A. hiPSC culture and differentiation

Human iPSC line, BC1,1-2 was dissociated into a single cell suspension and seeded at a density of 150,000 cells per fibronectin micropatterned coverslip in alpha MEM media, 10% fetal bovine serum, and 0.1 mM β-mercaptoethanol. Media was changed daily.

B. Immunofluorescence

Cells were fixed at 1, 2, and 5 days and prepared for immunofluorescence as previously described[1]. Briefly, fixed cells were blocked in 1% bovine serum albumin, treated with 0.1% Triton-X (Sigma-Aldrich), and incubated with mouse anti-human Tra-1-81 (BD Biosciences), followed by anti-mouse FITC (Sigma) and Phalloidin Alexa-546 (Life Technologies), and DAPI (Roche Diagnostics). The immunolabeled cells were examined using a fluorescent microscope (Olympus BX60).

C. Computational Image Analysis and Cell Quantification

All images were processed using a custom written Matlab algorithm incorporating the imaging processing toolbox. Prior to enumerating Tra-1-81 positive cells, individual JPEG files were preprocessed via background subtraction. Total nuclei was then pinpointed by local pixel intensities within a defined regional array and counted. Finally, individual channels were masked as binary images subsequent to excluding extraneous pixel values and co-localized with DAPI images for quantifying the percentage of stem to non stem cell populations.

D. E-cadherin Anti-body Blocking Experiment

To elucidate the role of cell-cell interactions in stem cell differentiation kinetics within confined geometric domains, 50 μg/mL of anti-E-cadherin antibody (clone 67A4; Millipore) was incubated with freshly dissociated hiPSCs for two hours. As evidence suggests disruption of E-cadherin signaling leads to increased stem cell death[2], 500,000 cells were subsequently seeded onto the micropatterns, cultured for an additional 24 hours[3] and prepped for immunofluorescence as described above.

II. MATHEMATICAL MODEL DETAILS

A. Stochastic two-species growth model: effects of interconversion and competition

The experimental system in the main text can be modeled by a stochastic birth-death process[4–6] with den-

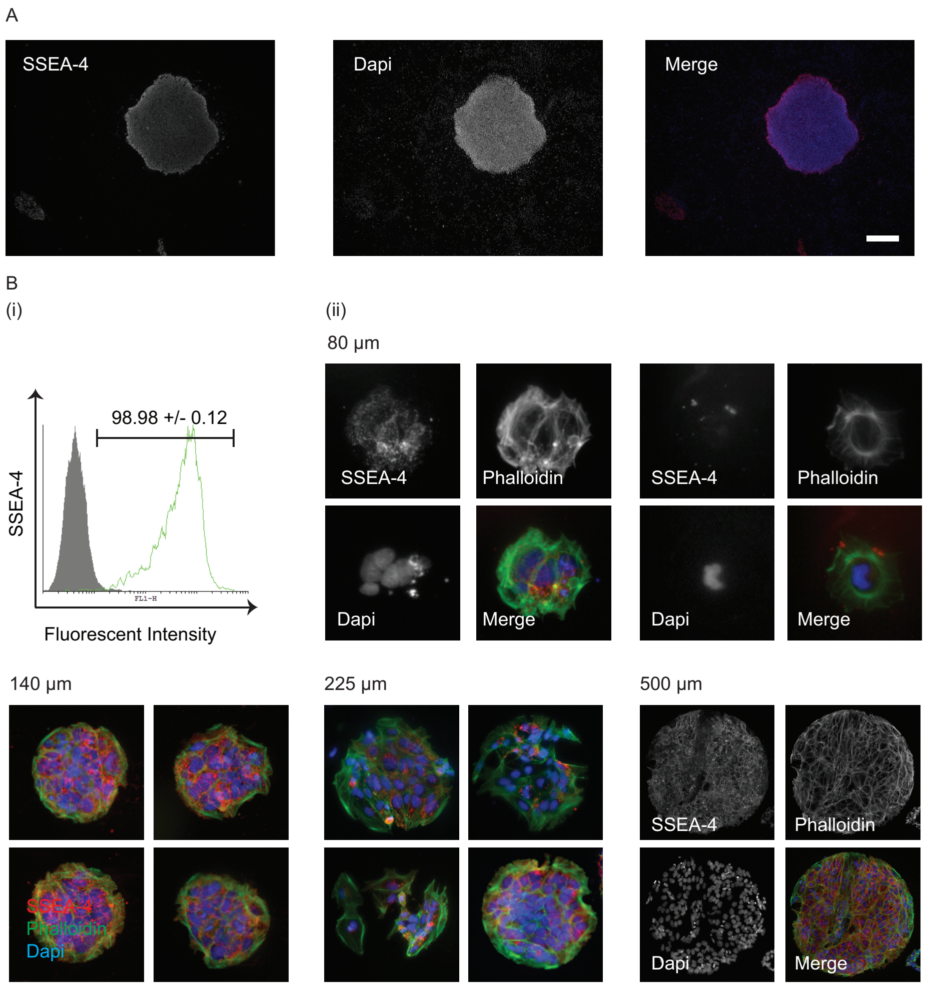

FIG. S2. Confirmation of stem cell pluripotency using stage-specific embryonic antigen-4 marker expression (SSEA-4). (A) Immunofluorescence imaging shows pluripotent colonies with SSEA-4 expression with nuclei staining. Scale bar 1mm. (B) Quantification of SSEA-4 expression via flow cytometry analysis (i) and day 1 SSEA-4 immunofluorescnce 24 hours post micropattern seeding shows pattern specific organization of stem and non-stem populations (ii).

sity dependent rates for symmetric cell division $u_S = v_S - \gamma_S(n_S - 1 + n_D)$ and $u_D = v_D - \gamma_D(n_D - 1 + n_S)$ for species S (stem cells) and D (differentiated cells), respectively. Here, v_S and v_D are intrinsic rates of cell proliferation in very sparse cell cultures while γ_S and γ_D are crowding coefficients. In this system, there is also potentially cell loss or cell death, mostly from cells detaching from substrate. We model cell loss with rates w_S and w_D for stem and differentiated cells. Since cell loss comes from mostly cells detaching from the substrate, and this rate is similar for stem and differentiated cells, i.e., $w_S \sim w_D$. Note that the rate constants of cell division for species S and D depend on total population size $n_S + n_D$. We will call this stochastic model as Model 1. Also, linear expressions for rate constants of cell proliferation make sense as long as the rates are positive. For large cell abundances ($n_S \gg 1$, or/and $n_D \gg 1$) we will assume that $u_S = 0$ and $u_D = 0$ when the linear expression result in negative rates. Model 1 predicts that the net growth diminishes as the population grows (and cell density increases, as the space for growth is limited) and can be used to describe homeostatic populations. In this model, all cells divide *slower* in the more crowed environment and the population reaches steady state.

In addition to cell proliferation, stem cells S can differentiate, i.e., convert to species D with rate r using several different possible mechanisms. We can consider three scenarios: (a) direct conversion: $S \to D$, (b) asymmetric cell division: $S \to S + D$, and (c) symmetric cell division: $S \to D{+}D$. Note that for mechanism (a) the total number of cells does not change while for mechanisms (b) and (c) the total number of cells increases. Therefore, in this paper, we focus on (a). (c) is not very different from (a) if we consider cell division and then conversion as two success steps. Therefore, results only vary quantitatively. The form of the differentiation probability and main conclusions of the paper remain the same. For (b), the number of stem cells cannot decrease, which is not

what we observe in the experiment. Therefore, we eliminate (b) from our consideration.

In the experiments, we discover that the rate of stem cell differentiation is a function of the composition of a population. We expect that rate of differentiation is a function of fractions of stem and nonstem cells in a population. In general, we can expand the rate as a polynomial function of stem cell fraction, $\chi = n_S/(n_S + n_D)$. To second order, the rate of cell differentiation can be written as

$$r = r_0 - f_1 \frac{n_S}{(n_S + n_D)} - f_2 \left[\frac{n_S}{(n_S + n_D)} \right]^2 . \qquad (1)$$

The fraction of differentiated cells is $1-\chi$, therefore writing r as a function of differentiated cell fraction will yield a similar expression. As previously, this expression for stem cell conversion rate is valid as long as it gives the non-negative rates of differentiation. For the range of parameters where expression becomes negative we assume that $r = 0$.

Now let us consider the set of stochastic equations describing the time dependence of cell number distribution of S and D). Let $P(k, m, t)$ be the probability to find $n_S \equiv k$ stem cells and $n_D \equiv m$ differentiated cells at time t. The general stochastic master equations are

$$\begin{aligned} \frac{dP(k,m,t)}{dt} &= U_{k-1,m,S} P(k-1, m, t) \\ &+ U_{k,m-1,D} P(k, m-1, t) \\ &+ W_{k+1,m,S} P(k+1, m, t) \\ &+ W_{k,m+1,D} P(k, m+1, t) \\ &+ R_{k+1,m-1} P(k+1, m-1, t) \\ &- (U_{k,m,S} + U_{k,m,D} + W_{k,m,S} + W_{k,m,D} + R_{k,m}) \\ &\times P(k, m, t) \end{aligned} \qquad (2)$$

with cell division rates based on cell density dependent rate constants

$$\begin{aligned} U_{k,m,S} &= v_S k - \gamma_S (k - 1 + m) k \\ U_{k,m,D} &= v_D m - \gamma_D (m - 1 + k) m \end{aligned} \qquad (3)$$

for $k = 0, 1, \ldots$ and $m = 0, 1, \ldots$, and loss rates based on fixed rate constants

$$\begin{aligned} W_{k,m,S} &= w_S k \\ W_{k,m,D} &= w_D m \end{aligned} \qquad (4)$$

for $m = 0, 1, \ldots$ and $k = 0, 1, \ldots$. For states (k, m) where the expressions in Eq. (3) become negative, U is replaced by zero. The rate of stem cell differentiation is given by *piecewise* function

$$R_{k,m} = r_0 k - f_1 \frac{k}{(k+m)} k + f_2 \left[\frac{k}{(k+m)} \right]^2 k \qquad (5)$$

For states (k, m) of the system where the rates expressed by Eq. (5) become negative, R is replaced by zero. The coefficients f_1 and f_2 for stem cells may have different signs.

From Eqs. (3) it follows that

$$U_{0,k,S} = U_{m,0,D} = W_{0,k,S} = W_{m,0,D} = 0 \qquad (6)$$

The infinite set of Eq. (2) should be supplemented by

$$P(-1, j, t) = P(j, -1, t) \equiv 0 \qquad (7)$$

for $j = 0, 1, \ldots$. Practically, we solve the finite set of stochastic master equations by introducing the truncation size K. The set of rates at m or k equal to K is defined as

$$U_{K,j,S} = U_{j,K,D} = 0 \qquad (8)$$

$$W_{K+1,j,S} = W_{j,K+1,D} = 0 \qquad (9)$$

for $j = 0, 1, \ldots, K$ and also

$$R_{K,j} = 0 \qquad (10)$$

$$R_{K+1,j} = W_{j,K+1,D} = 0 \qquad (11)$$

for $j = 0, 1, \ldots, K + 1$. Also instead of Eq. (7) in numerical computations we use the following expression for rates:

$$u_{-1,j,S} = u_{j,-1,D} = 0 \qquad (12)$$

for $j = 0, 1, \ldots, K$.

$$R_{j,-1} = 0 \qquad (13)$$

for $j = 0, 1, \ldots, K+1$. Eqs. (12) and (13) are not related with truncation size K, they just express that no transitions are possible from/to non-existing states $(-1, j)$, or $(j, -1)$ that are formally present in stochastic equations for $P(0, j, t)$ and $P(j, 0, t)$.

Eqs. (8-11) expresses the condition that the population size cannot grow larger than $n_S = K$ and $n_D = K$. We used $K = 100$ as the truncation size in our computations for describing the population dynamics of stem/nonstem cells on small micro patterns.

The set of stochastic master equations can be solved for a given initial conditions $P(k, m, 0) = P_0(k, m)$. In the experiments, the micropatterns initially are plated by stem cells with some constant cell density. In this case differentiated cells are not present at $t = 0$ and the initial distribution of stem cells is expected to be a Poisson distribution

$$P(k, 0, 0) = \lambda^k e^{\lambda} / k! \qquad (14)$$

and $P(k, m > 0, 0) = 0$, where λ is the initial average number of stem cells on a pattern of a given size.

We solve the system of stochastic master equations numerically and obtain at each time t the probabilities $P(k, m, t)$ of states with k stem and m differentiated cells.

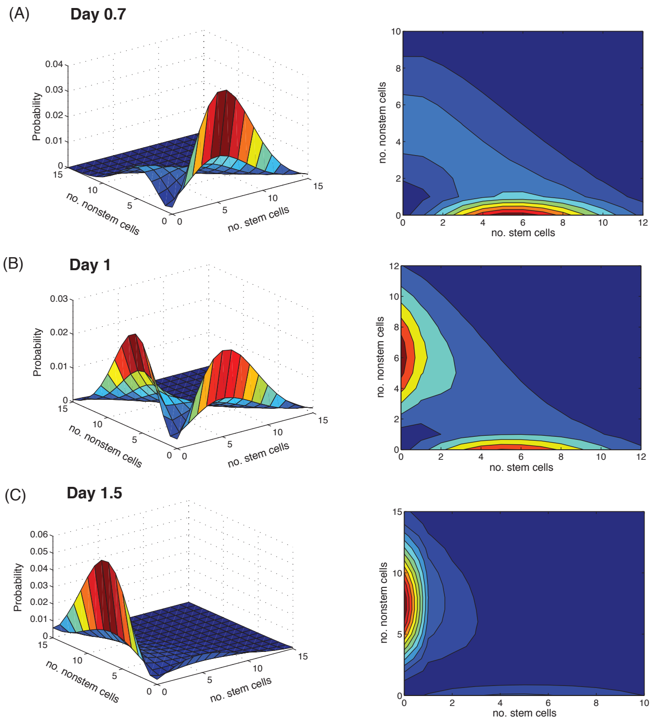

FIG. S3. Computed probability distribution of stem and differentiated cells from the stochastic master equation, Eq. (2). (A) After 0.7 day. (B) After 1 day, and (C) After 1.5 days. The distribution evolves towards 100% differentiated cells. The average number of stem and differentiated cells, and number fluctuations are also shown in Fig. 2 of the main text.

The probabilities of states fully define the state of a system. For instance, they allow computing any moments of joint probability distribution. The average number of stem and differentiated cells at time t are given by

$$\langle n_S \rangle = \sum_{k=0}^{K} \sum_{m=0}^{K} k P(k,m,t)$$
$$\langle n_D \rangle = \sum_{k=0}^{K} \sum_{m=0}^{K} m P(k,m,t) \tag{15}$$

and the variances (squares of standard deviations) and covariance are obtained as

$$\sigma_S^2 = \sum_{k=0}^{K} \sum_{m=0}^{K} k^2 P(k,m,t) - \langle n_S \rangle^2$$
$$\sigma_D^2 = \sum_{k=0}^{K} \sum_{m=0}^{K} m^2 P(k,m,t) - \langle n_D \rangle^2$$
$$\sigma_{S,D} = \sum_{k=0}^{K} \sum_{m=0}^{K} k\, m P(k,m,t) - \langle n_S \rangle \langle n_D \rangle \tag{16}$$

The average numbers of stem $\langle n_S \rangle$ and differentiated $\langle n_D \rangle$ cells along with their standard deviations σ_S and σ_D are compared with experimentally observed values (Fig. 2 main text). For a state (k,m) stem cells fraction is $k/(k+m)$. Hence, the probability to obtain the fraction $\chi(t)$ of stem cell in a population at time t is given by summing up of all $P(k,m,t)$ with $k/(k+m) = \chi$. The one-dimensional distribution $\rho_S(\chi,t)$ is normalized since it is derived from normalized two-dimensional distribution $P(k,m,t)$.

$$\rho_S(\chi,t) = \sum_{k=0}^{K} \sum_{m=0}^{K} P(k,m,t) \delta(\chi - k/(k+m)) \tag{17}$$

From the master equation, we computed two-dimensional probability distributions for the model that assumes appreciable dependence of the conversion rate on stem cell fraction. Fig. S2 depicts the joint probability distribution for numbers of stem and nonstem cells at $t = 1$, 2 and 5 days. This distribution has two distinct maxima. The composition of cell colonies in collection of micropatterns described by this distribution is highly heterogeneous corresponding to populations highly enriched by stem cells or nonstem cells. The one-dimensional stem cell fraction probability distribution is bimodal with sharp maxima around $\chi = 0$ and $\chi = 1$. In contrast, we the differentiation probability r as a constant, independent of χ, we always find a single peak in the cell number distribution (Fig. S3).

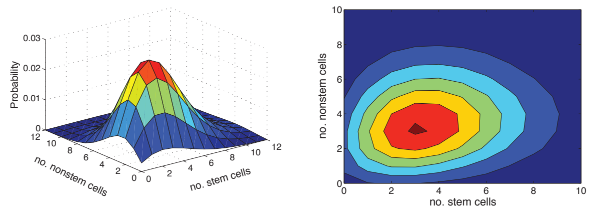

FIG. S4. Computed probability distribution of stem and differentiated cells from the stochastic master equation (Eq. (2) with a constant stem cell differentiation rate, r, independent of χ). The distribution is always unimodal.

B. Modeling stem cell differentiation dynamics on small micro pattern

Given the modeling framework, we fit the model parameters to experimental data. The procedure of finding the best parameters of the models is based on the minimization of an error function by the Monte Carlo (MC) method. The error function is composed of two parts and represents the sum of deviations of theoretical data from experimental observations. The first part shows how the average numbers of stem and nonstem cells on micro pattern agree with experimental observations. The second part measures the deviations of theoretical probability distribution function for stem cell fraction for a group of micropatterns of a given size from experimental results.

In our case the error function reads

$$g_1 = \sum_{i=1}^{K} [\langle n_S \rangle(t_i) - n_S^0(t_i)]^2 + [\langle n_D \rangle(t_i) - n_D^0(t_i)]^2$$

$$g_2 = \sum_{i=1}^{K} \sum_{j=0}^{N} [\rho_S(\chi_j, t_i) - \rho_S^0(\chi_j, t_i)]^2$$

$$E = \omega_1 g_1 + \omega_2 g_2 \quad (18)$$

where E is the total error function and $g_i (i = 1, 2)$ are its parts; $\langle n_S \rangle$ and $\langle n_D \rangle$ are theoretical numbers of stem and nonstem cells at time t_i ($K = 3$ is the number of time points at which the cell colonies were quantified), and n_S^0 and n_D^0 are experimental values of average numbers of stem and nonstem cells for micropatterns of a given size. $\rho_S(\chi_j, t_i)$ and $\rho_S^0(\chi_j, t_i)$ are theoretical and experimental values of probability density function for stem cell fraction evaluated at time point t_i.

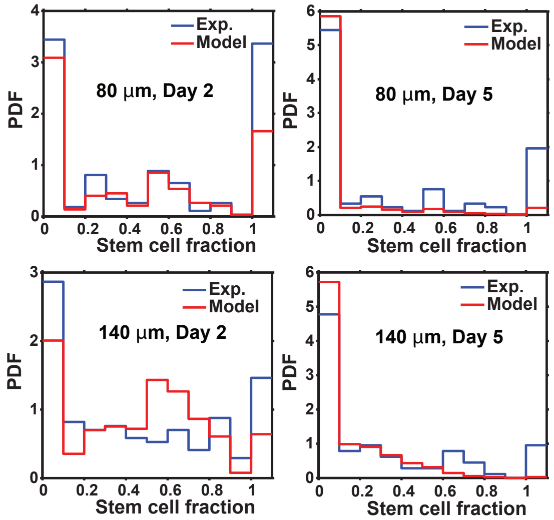

FIG. S5. Computed distribution of stem cell fraction for 80 and 140μm micro patterns after 2 and 5 days. The model (red lines) is able explain the experimental data (blue lines) with a single set of parameters. The parameters that best explain the data are given in Table I.

The algorithm of finding the best parameters using the MC method is as follows: (1) We start with an initial guess of parameters in the model: intrinsic proliferation rates, crowding coefficients and cell death rates for stem and nonstem cells, respectively, and also parameters describing the rate of differentiation as a function of local fraction of stem cells in a population; (2) All parameters are changed randomly by small values; (3) We solve numerically the system of stochastic master equations for initial guess and perturbed parameters; (4) If the trial function g is smaller for changed set of parameters and all these parameters are consistent with constraints, the new parameters are accepted otherwise the parameters remain intact. The new or unchanged parameters are perturbed again (see item 2). Thus, we accomplish the constrained search of parameters, so the new set of parameters that diminishes the trial function is accepted only if all these parameters are consistent with constraints. We put the following constraints on parameters: all crowding coefficients that describe the proliferation rates of cells on the total population size should be positive and also the intrinsic proliferation rates should exceed some pre-set values. The obtained best fits are checked by starting with other initial guesses to see convergence to the same set of final parameters. We complete the procedure of finding the best parameters when the trial function practically ceases to change and theoretical description of experimental data remains practically the same. The parameters of the model found by minimization of error function E by the MC method is presented in Table I.

STEM CELL DIFFERENTIATION ON LARGE MICRO PATTERNS: THE MODEL WITH COMPARTMENTS

The model with compartments is used to capture the main experimental observations on stem cells differentiation in micro patterns with large sizes. A large micro pattern is considered as a combination of small compartments. The probability of stem cell conversion is expected to be a function of local composition of a single compartment rather than the global composition of a population in the whole micropattern. The functional dependence of conversion rate vs. stem cell fraction is taken identical to cell differentiation on small micropatterns. Cell motility is taken into account by introducing stochastic hopping between compartments. Stem and nonstem cells can leave their original compartment and occupy the neighboring one. The hopping rate of a cell from one compartment to another one is k_h, which is related to the effective diffusion constant of cells as

$$k_h = \frac{D}{L^2} \quad (19)$$

where $L = 100\mu$m is the compartment size.

We use a stochastic simulation algorithm to compute dynamics in the compartment model. For a given time interval, δt, we consider the probability of escaping the current state, $e^{-K\delta t}$, where K is the sum of all possible rates of escape from the current state. For example, we include sums of all rates of proliferation $u_{S,D}$ and cell loss $\delta_{S,D}$ for each cell in all compartments in K. We also include the rate of stem cell differentiation, r, and rates of diffusion for each cell, k_h. We then use an acceptance criterion $R = \min[1, e^{-K\delta t}]$, where R is a random number. To determine the actual stochastic event, we select another random number and select from the list of all possible events weighted by their rates. The parameters specifying proliferation and cell loss in each compartment are exactly the same as 140μm parameters in Table 1. Using these parameters we obtained 3000 trajectories for a

TABLE I. Fitted model parameters of the stochastic differentiation model on smaller (80 and 140μm) micro patterns. The Initial average number of stem cells is λ, whose distribution is given by Eq. (14).

Parameter (day^{-1})	80 μm	140 μm	Equation
v_S	0.37	0.66	3
v_D	0.18	0.13	3
γ_S	4.5 x 10^{-4}	0.6 x 10^{-3}	3
γ_D	1.9 x 10^{-2}	0.7 x 10^{-2}	3
w_S	0.025	0.025	4
w_D	0.11	0.08	4
r_0	1.5	1.5	5
f_1	1.8	2.17	5
f_2	0.7	1.09	5
λ	1.5	3	14

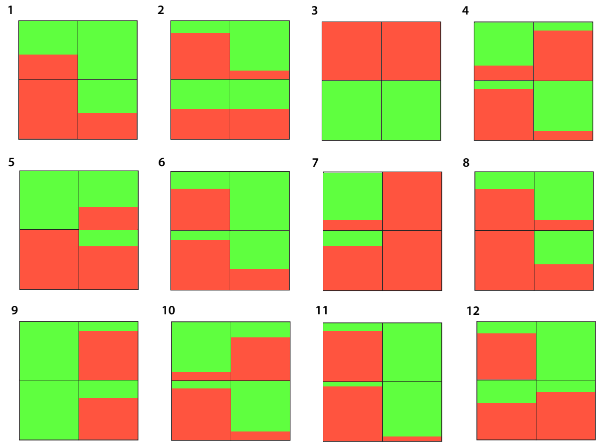

FIG. S6. Twelve examples of simulated configuration for the 4-compartment model after 1 day. The configurations are close to the average observed stem cell fraction of $\chi = 0.44$. The amount of red or green in each compartment corresponds to the fraction of stem and differentiated cells. The individual compartments can still be dominated by either stem or differentiated cells.

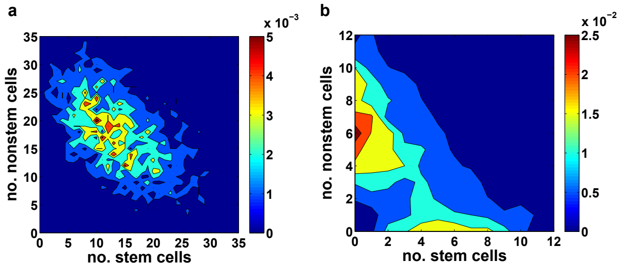

FIG. S7. Computed 2-dimensional probability distribution of numbers of stem and differentiated cells at t = 1 day for a pattern with 4 100μm compartments. (a) The summed distribution in the whole pattern comprised four compartments; (b) A distribution from an individual compartment.

micro pattern comprised four compartments. The initial number of stem cell follows the Poisson distribution with an average $\lambda = 24$, mimicking the experimental setup when the micropatterns are seeded by stem cells with particular density but the exact number of cells in each micropattern is not precisely controlled.

The results of these model MC simulations show that at day 1, the average fraction of stem cells is $\chi = 0.44$, which is close to experimentally observed values for 225 μm and 500 μm micropatterns. The analysis of trajectories shows that either mostly stem cells $\chi \simeq 1$, or mostly differentiated cells $\chi \simeq 0$ are present in individual compartments while the distribution of stem cell fraction in a whole micropattern is unimodal and features a maximum near the average χ. Fig. S6 shows the two-dimensional probability distribution of numbers of stem and differentiated cells in the whole pattern comprised four compartments at $t = 1$ day. Cell motility is taken into account. The total distribution summed for all compartments reveals one maximum that is also consistent with unimodal one-dimensional distribution of stem cell fraction where the maximum is close to average value. The individual compartment distribution can still exhibit a bimodal distribution.

MEAN POPULATION MODEL FOR STEM CELL DIFFERENTIATION

Here we focus briefly on difference between stochastic and mean population model models for describing the population dynamics of stem cells. The mean population model considers only the time evolution of average number of species derived from expressions for rate constants. These models are often used in chemical kinetic equations. For our system, equations that describe the time evolution of average numbers of stem and differen-

tiated cells can be written as

$$\frac{d\langle n_S\rangle}{dt} = u_S\langle n_S\rangle + R\langle n_D\rangle - w_S\langle n_S\rangle$$
$$\frac{d\langle n_D\rangle}{dt} = u_D\langle n_D\rangle - R\langle n_D\rangle - w_D\langle n_D\rangle \quad (20)$$

with rate constants expressed through the average numbers of species (X = S, D)

$$u_X = v_X - \gamma_X(n_S + n_D)$$
$$R = r - f_1 n_S/(n_S + n_D) - f_2[n_S/(n_S + n_D)]^2 \quad (21)$$

This simple system of two differential equations can be solved numerically. Comparison between this mean field approach and the full stochastic model for average stem and differentiated cells are shown in Fig. S4. Note that the mean field model cannot compute probability distributions of stem cell fractions from our data. The mean field model also does not consider possible population fluctuations away from the average. Since in our system the cell number is small, fluctuations are substantial, and affect the mean population.

Alternatively, starting with the full stochastic model of Eq. (2), we can perform a cumulant expansion by considering moments such as

$$\langle n_S^M\rangle(t) = \sum_{k,m} k^M P(k,m,t)$$
$$\langle n_D^N\rangle(t) = \sum_{k,m} m^N P(k,m,t) \quad (22)$$

and mixed moments

$$\langle n_S^M n_D^N\rangle(t) = \sum_{k,m} k^M m^N P(k,m,t) \quad (23)$$

For instance, for $M = N = 1$, the above Eq. (22) is equivalent to the average populations in Eq. (15). The time evolution of the complete set of moments of the distribution $P(k,m,t)$ can be derived from the full master equation. For instance, for the average populations

$$\frac{\partial\langle n_S\rangle}{\partial t} = \sum_{k,m} k \frac{\partial P(k,m,t)}{\partial t}$$
$$\frac{\partial\langle n_D\rangle}{\partial t} = \sum_{k,m} m \frac{\partial P(k,m,t)}{\partial t} \quad (24)$$

Substituting the right hand side of Eq. (2) for $\partial P/\partial t$ will give a complicated function of higher moments of P. Note that this set of coupled moment equations are in principle exact. Approximations can be made by cutting off the moment equations and introducing a closure relation. The typical population models of Eq. (20) is not equivalent to the cumulant expansion, even if we cut the expansion off at first order and neglect any terms involving higher moments of P.

Comparisons between Eq. (20) and the full stochastic equation solutions are shown in Fig. S3. The mean-field

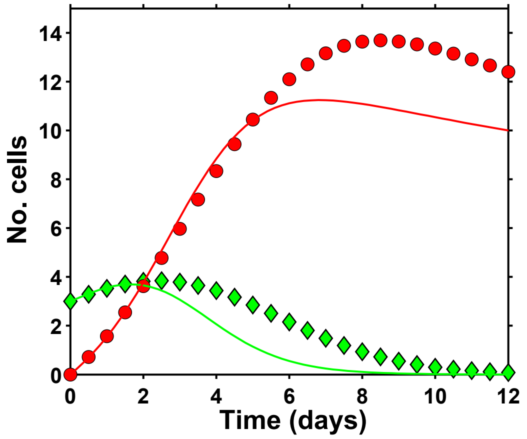

FIG. S8. Comparison between the mean population model of Eq. (20) (solid lines) with the stochastic master equation (dots). Red is the number of differentiated cells and green is the number of stem cells. Since the number of cells is small, the mean population model does not agree with the full stochastic solution.

model with composition dependent conversion rate (Eq. (21) is significantly different from the stochastic model. For a constant stem cell conversion rate, mean field model can agree with the full stochastic solution. Of course, the mean field model cannot obtain distributions of stem cell fractions such as shown in Fig. 1 of the main text.

[1] Abaci HE, Shen YI, Tan S and Gerecht S. (2014) Recapitulating physiological and pathological shear stress and oxygen to model vasculature in health and disease. Sci, Rep. 4:4951.

[2] Li L, Bennett SAL, and Wang L (2012) Role of E-cadherin and other cell adhesion molecules in survival and differentiation of human pluripotent stem cells. Cell adh. Migr., 6:59-73.

[3] Dang SM, Gerecht-Nir S, Chen J, Itskovitz-Eldor J, and Zandstra PW (2004) Controlled, scalable embryonic stem cell differentiation culture. Stem Cells, 22:275-282.

[4] Novak, M (2006) Evolutionary dynamics: Exploring the equations of life. Belknap Press.

[5] Sun SX, Lan G and Atilgan E (2008) Stochastic modeling methods in cell biology. Meth. Cell Biol. 89:601-621.

[6] McQuarrie DA (1967) Stochastic approach to chemical kinetics. J. Appl. Prob. 4:413478.
